# Supplementary material for: Structural identification of a selectivity filter in CFTR
Source: Proc Natl Acad Sci U S A. 2024 Feb 21;121(9):e2316673121. doi: 10.1073/pnas.2316673121 (PMC10907310; doi:10.1073/pnas.2316673121)
Supplement: Supplementary file 1 — Appendix 01 (PDF) [file pnas.2316673121.sapp.pdf]

Supplementary Information for

Structural Identification of a Selectivity Filter in CFTR

Jesper Levring[1] & Jue Chen[1,2,*]

[1]Laboratory of Membrane Biology and Biophysics, The Rockefeller University, New York, NY 10065, USA.

[2]Howard Hughes Medical Institute, The Rockefeller University, 1230 York Ave, New York, NY, USA.

*To whom correspondence should be addressed: juechen@rockefeller.edu.

This PDF file includes:

Figures S1 to S4

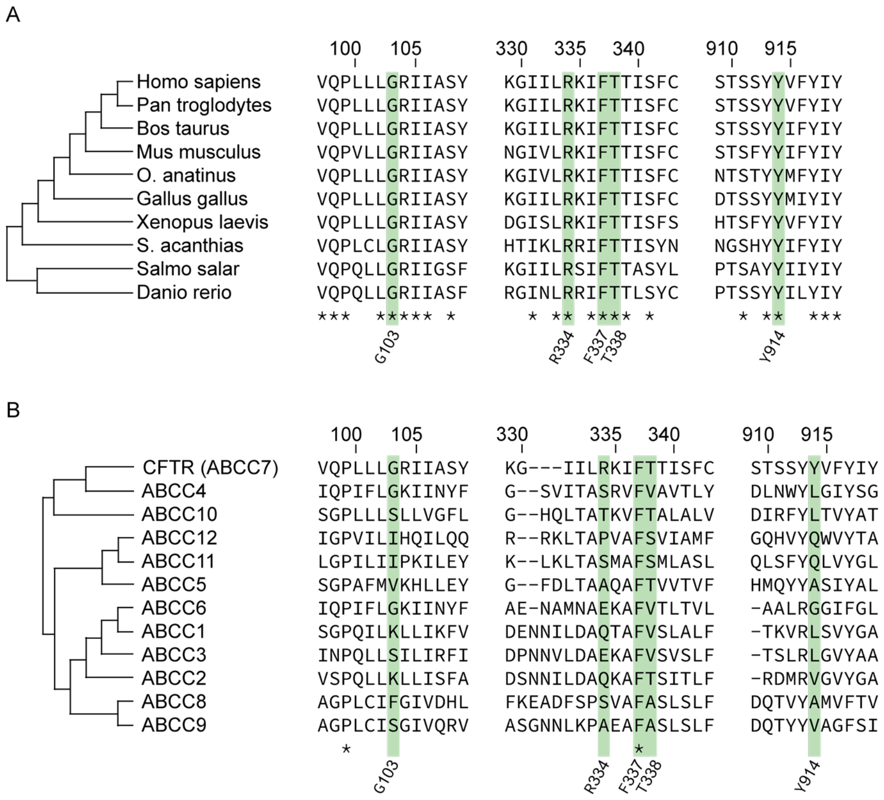

Figure S1 | Sequence alignment of regions contributing to chloride coordination. (**A**) Alignment of selected CFTR orthologues. (**B**) Alignment of human ABCC paralogues. Residues that coordinate chloride in CFTR are highlighted in green. Asterisks indicate invariance across the aligned sequences.

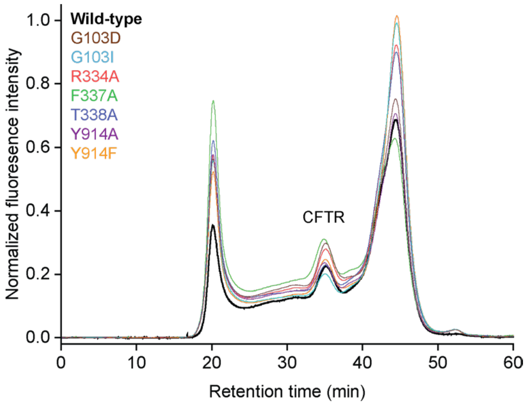

Figure S2 | Fluorescence size-exclusion chromatograms of C-terminally GFP-fused CFTR S_{filter} variants.

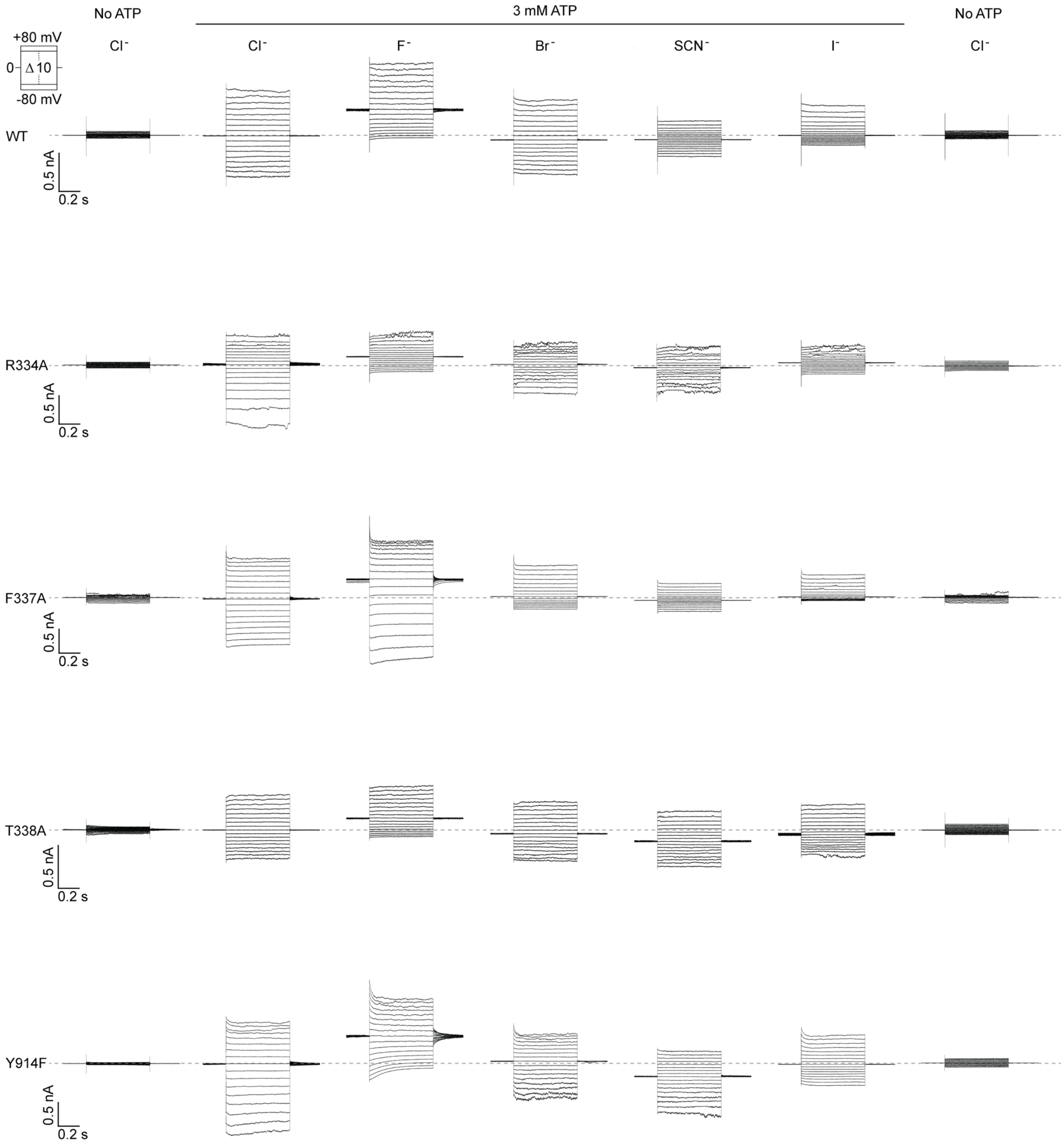

Figure S3 | Biionic potential measurements for selectivity filter variants. Example voltage families for PKA-phosphorylated CFTR variants under biionic conditions in inside-out excised patches. The patch pipette contained 150 mM chloride, and the perfusion solution contained 150 mM of the indicated anion. 3 mM ATP was included in the perfusion solution where indicated. ATP-free measurements were performed at the beginning and end of each patch to ensure absence of CFTR-independent anion leak.

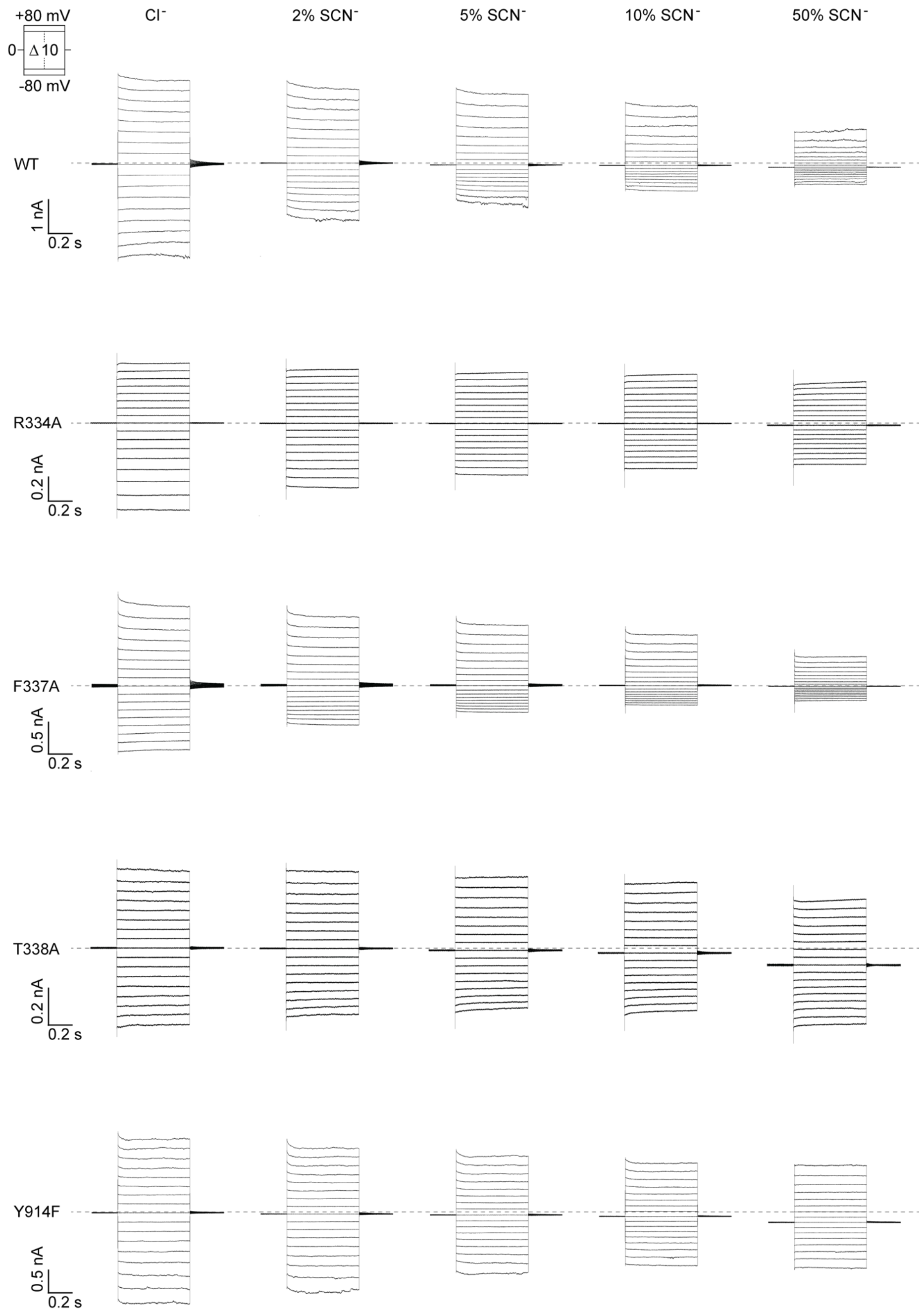

Figure S4 | Open channel block of selectivity filter variants by thiocyanate. Example voltage families for PKA-phosphorylated CFTR variants with the indicated mole fractions of thiocyanate. The patch pipette contained 150 mM chloride, and the perfusion solution contained a mixture of chloride and thiocyanate. 3 mM ATP was used in the perfusion solution for all experiments.
